# Histopathological evaluation of ocular microsporidiosis by different stains

**DOI:** 10.1186/1472-6890-6-6

**Published:** 2006-06-23

**Authors:** Joveeta Joseph, Geeta K Vemuganti, Prashant Garg, Savitri Sharma

**Affiliations:** 1 Jhaveri Microbiology Centre, Hyderabad Eye Research Foundation, L.V.Prasad Eye Institute, L.V.Prasad Marg, Banjara Hills, Hyderabad – 500 034, India; 2 Ophthalmic Pathology Services, Hyderabad Eye Research Foundation, L.V.Prasad Eye Institute, L.V.Prasad Marg, Banjara Hills, Hyderabad – 500 034, India; 3 Cornea and Anterior Segment Services, Hyderabad Eye Research Foundation, L.V.Prasad Eye Institute, L.V.Prasad Marg, Banjara Hills, Hyderabad – 500 034, India

## Abstract

**Background:**

There is limited data on comparing stains in the detection of microsporidia in corneal biopsies. Hence we wanted to evaluate various stains for their ability to detect microsporidia in corneal tissue sections.

**Methods:**

Four cases diagnosed with microsporidiosis on Hematoxylin and Eosin and Periodic Acid Schiff's stained sections of the corneal button between January 2002 and December 2004, were included. Further sections were prospectively stained with calcofluor white, Gram, Giemsa, Masson's trichrome, acridine orange, Gomori's methenamine silver, Gram's chromotrope and modified acid fast stain. The stained sections were analyzed for the spore characteristics in terms of size, shape, color contrast, cell wall morphology, waist band in cytoplasm and ease of detection.

**Results:**

All sections showed microsporidial spores as 3 – 5 μm, oval bodies. 1% acid fast, Gram's chromotrope and GMS stains provided a reliable diagnosis of microsporidia as diagnostic waist band could be identified and good contrast helped distinguish the spores from inflammatory debris.

**Conclusion:**

Considering the ease of performance, cost effectiveness and rapidity of the technique, 1% acid fast stain and Gram's chromotrope stain are ideal for the detection of microsporidia.

## Background

Microsporidia is a nontaxonomic designation used to refer to a group of obligate intracellular protists belonging to the phylum Microspora [1]. In humans, microsporidia are opportunistic pathogens that cause gastrointestinal, sinus, pulmonary, muscular, renal and ocular diseases. Microsporidia is an important cause of morbidity, and occasionally, mortality in patients with AIDS [2]. Two distinct clinical entities of this disease in the eye have been described: deep corneal stromal infection and superficial keratoconjunctivitis [2]. In most of cases diagnosis of microspoirdiosis could be made by examination of biopsy and autopsy specimens. Examples include cornea, conjunctiva, skeletal muscle, small and large intestine, liver, gallbladder, bile duct, pancreatic duct, omentum, kidney, ureter, bladder, prostate, and trachea or bronchi [2]. It is important for pathologists and microbiologists to be knowledgeable regarding the identification of these agents in biopsy, autopsy and cytology specimens.

Light microscopic examination remains the standard test for the diagnosis of microsporidiosis, and considerable progress has been made in the development of additional stains for the diagnosis of these organisms. They appear as oval to piriform spores measuring 2–7 μm in length and 1.5 to 5 μm in width. Most of the stains described in various reports are based on observations on cytologic preparations [2,3]. The stains that are used on cytologic preparations may not show similar results in biopsy specimens, due to inherent processing artifacts produced and background staining.^3 ^The various stains evaluated on tissue sections include chromotrope 2R modified trichrome, haematoxylin and eosin, Gram and Giemsa stains on jejunal biopsies [4]. The spores are appreciated as small refractile bodies in haematoxylin and eosin stain with an unstained area of spore wall, larger than their actual size in modified Warthin-Starry stain [5] and are Gram positive, although some variability in the intensity of staining can be noted [3]. Evaluation of Fungifluor, Calcofluor White, and Fungiqual A fluorochrome stains in biopsy imprints and paraffin biopsy sections [6] revealed all three stains to be rapid and sensitive for the detection of microsporidial spores. More recently, Gram's chromotrope staining of microsporidial spores has been described by several laboratories as yielding good results, being a rapid diagnostic procedure that combines the properties of the Gram staining with those of Weber's chromotrope [7].

In the examination of histologic specimens, microsporidia can be overlooked, because they may evoke minimal or no inflammatory response depending on the type of tissue/host immune status [3]. They appear as pale staining, ill-defined oval structures will a clear halo due to unstained spore wall which could resemble the yeast forms. Though microsporidial keratitis has been reported by various groups [8-10], there is limited data comparing and evaluating various stains in the detection and confirmation of microsporidia in corneal biopsies. To familiarize ourselves with the morphological appearance of microsporidia, we performed various histochemical staining techniques on corneal tissues and also analyzed the advantages and disadvantages of the various procedures. The stains chosen in our study are routinely used in our laboratory for the diagnosis of bacterial, fungal and *Acanthamoeba *keratitis and hence we wanted to acertain the most useful stain for the diagnosis of microsoridia keratitis.

## Methods

Patients: The cases of microsporidial keratitis diagnosed at the L.V.Prasad Eye Institute, Hyderabad, India between January 1, 2002 and 31 December, 2004, that underwent penetrating keratopalsty were included in the study. The medical records of all cases were reviewed for the demographic data.

The corneal button of the cases following surgery were fixed in 10% buffered formalin and sent to the histopathological laboratory for routine processing. The sections were 3 to 5 microns thick. A diagnosis of microsporidial keratitis was made based on morphological features of the tissues stained with hematoxylin and eosin (H & E) stain and periodic acid-Schiff's (PAS) stain. Confirmation of these cases was done on 1% acid fast stain (Kinyouns modification of Ziehl Neelsen stain). The unstained permanent sections were further evaluated using Calcofluor white, Brown-Brenn Gram, Giemsa, Masson's trichrome, Acridine orange and Gomori's methenamine silver stain (GMS). All these stains have been described previously [11,12]. Giemsa stain was done with Diff Quick^®^, an equivalent of Wright/Giemsa stain (Bacto laboratories pty. ltd., Liverpool, NSW, Australia). The sections were also stained with Gram's chromotrope stain [5] at the Centres for Disease Control, Atlanta, USA and the results were included in this study.

For CFW staining, one drop each of 0.1% calcofluor white (Sigma, USA) and 0.1% Evans blue solution was added onto the section and a coverslip was placed on it. Similarly for acridine orange staining, one drop of 0.1% the stain was added onto the section and a coverslip placed on it. The wet preparation of calcofluor white was observed under the fluorescence microscope (BH2-RFC, Olympus) at × 500 magnification with cube U having filter combinations for the excitation spectrum region near 365 nm for DAPI stain. For acridine orange, it was observed with cube B having filter combinations for the excitation spectrum region near 470 nm for FITC stain. The remaining stains were observed at × 500 magnification with a bright field microscope.

## Results

In the study period, of the 2655 cases that underwent penetrating keratoplasty (PK), four were diagnosed histopathologically with microsporidial keratitis. Out of four corneal buttons, three were from patients diagnosed as stromal keratitis and one was from a corneal scar with descemetocele. The mean age of the patients was 37.25 ± 27.8 years (range 2 – 70 years) with male to female ratio being 1:1. While two patients gave a history of injury, the predisposing factor in the other two patients was unknown. The clinical features seen in microsporidial stromal keratitis are described in table 1. One patient had anterior stromal infiltrates, one presented with corneal opacity and two patients had deep stromal infiltrates with endothelial exudates. One patient who was on steroids, showed an initial response, but later perforation occurred and the patient was advised to undergo penetrating keratoplasty. None of these patients had a recurrence following PK.

**Figure 1:**
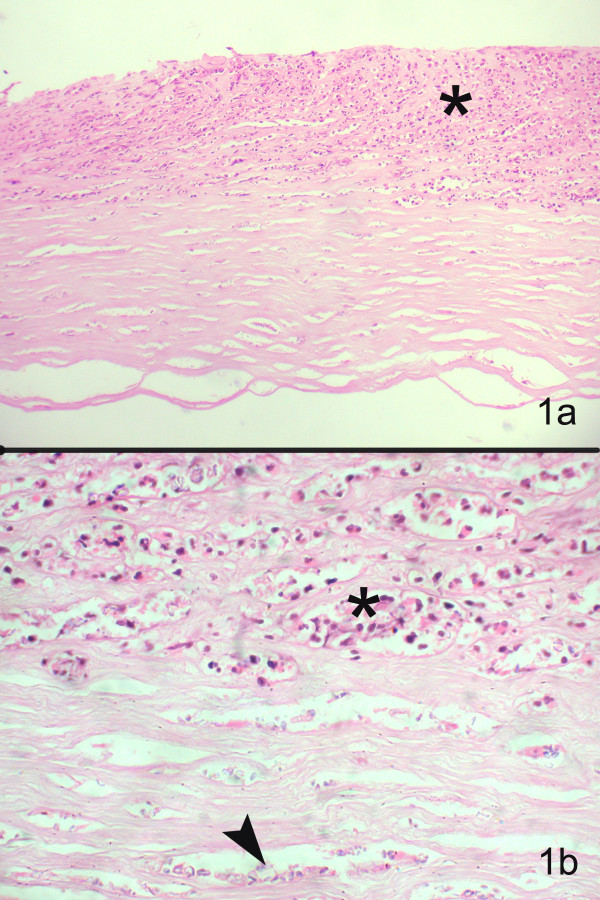
Section of the corneal tissue shows epithelial ulceration, with inflammatory infiltrates in the anterior two-thirds of stroma (hematoxylin & eosin, × 1100) (b) Higher magnification shows polymorphonuclear cells (asterix) and faintly stained, ill-defined oval dot like structures (arrow) between the corneal lamellae (Hematoxylin and Eosin stain, × 400)

**Figure 2:**
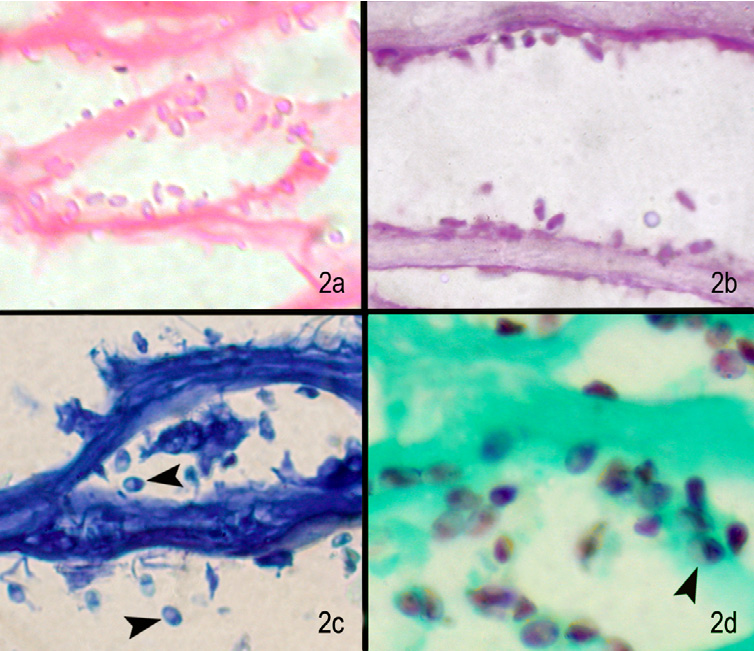
(a) Under higher magnification, the microsporidal spores are seen as pink oval structures (H & E stain, × 1000); (b) magenta pink oval structures in PAS stain (× 500), (c) deep blue oval structures with dark tip (arrow) in some spores Giemsa stain (× 500), (d) well defined brown oval spores with dark tip or band in Gomoris methenamine silver stain (× 500)

In the corneal button sections stained with haematoxylin and eosin, the epithelium was intact in two, showed edema in one and was ulcerated in one. Bowman's layer was destroyed in 3 out of 4 cases. There was moderate to severe stromal inflammation (Fig 1), at places forming microabscesses. The stromal inflammation consisted of polymorphonuclear cells, few mononuclear cells against a background of inflammatory debris. The inflammation was deep stromal and more in the pre-Descemet region, partly extending to the superficial layers. A few macrophages were also seen. Inflammation was absent in the corneal tissue from the patient diagnosed as corneal scar. All sections showed microsporidial spores as indistinct pale staining oval bodies, 2 – 3 μm wide and 3 – 5 μm in length (fig 2a) involving mostly the deep stroma, extending into the anterior layers in 2 cases. The unstained and faintly stained spores showed a thick-walled capsule that was unstained and birefringent on polarized light. Some forms of spores were difficult to distinguish against the nuclear debris in the background and could also be confused with yeast forms of fungi. However there was no evidence of budding in these spores. The morphological features observed under various stains are outlined in table 2.

### Periodic-acid Schiff's stain (PAS)

These organisms appeared pink to translucent in color. (Fig. 2b). The spores are oval to round in shape, unlike inflammatory cells which appear irregular. The spore morphology could not be appreciated and the diagnostic waist band could not be identified. Also there was very poor contrast making it difficult to distinguish these structures from the inflammatory debris.

### Giemsa stain

This stain also revealed microsporidia but with suboptimal morphology (Fig. 2c). The spores appeared blue in color though some remained unstained. There was poor differentiation from background and other inflammatory debris. In two cases the darkly stained belt could be identified, which helped in preliminary diagnosis.

### Gomori's methenamine silver stain (GMS)

These organisms appear oval to round, brown in colour (Fig. 2d) and the internal band girding the spore is easily spotted (arrow head). This is a distinguishing characteristic of the organism. This silver impregnation is highlighted against a green background which ruled out confusion with inflammatory cells.

### Calcofluor white stain

**Figure 3:**
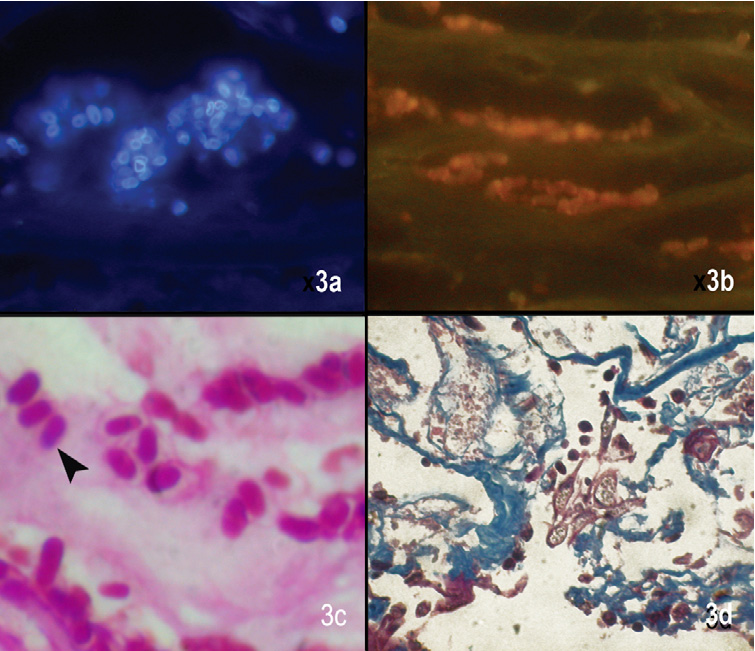
Microsporidal spores are seen as white fluorescent ring like structures in Calcofluor white stain (× 500), (b) dull oval orangish structures in Acridine orange stain (× 500), (c) oval well defined spores with a faint hollow around the spore in Gram stain (× 1000), (d) dark blue uniformely stained bodies in Masson's trichrome stain (× 500)

The organisms appeared diffusely fluorescing with fluorescence intensities depending on the maturity of the spore (Fig. 3a). In some cases the spore wall alone appeared fluorescing with a lot of background "noise". Though the spore morphology could be appreciated, the internal structures could not be seen. These spores could be mistaken for yeast cells, but the absence of budding can confirm a diagnosis of microsporidiosis. Background fluorescence interfered with the clear detection of the spores. Inflammatory cells do not fluoresce and thus can be easily differentiated.

### Acridine orange

The spores appeared oval, refractile with a orange fluorescence (Fig. 3b). There was no clear demarcation from the background. The spores were either scattered or highly clustered within the cytoplasm of occasional epithelial cells. Here too the spores could be mistaken for yeast cells, but budding, a common characteristic of yeast cells, is absent in microsporidial spores

### Brown-Brenn gram stain

The spores appeared gram variable and plenty of granular or inflammatory debris was seen. There was poor contrast (Fig. 3c), which made viewing of the spores difficult. The diagnostic waist band of these spores could not be appreciated.

### Masson's trichrome stain

The spores appeared deep red to black against a blue background, but these could be mistaken for other granular artifacts like nuclear debris which also take up the stain (Fig. 3d). This made identification of the spores difficult and the internal morphology could not be appreciated.

### 1% acid fast stain

**Figure 4:**
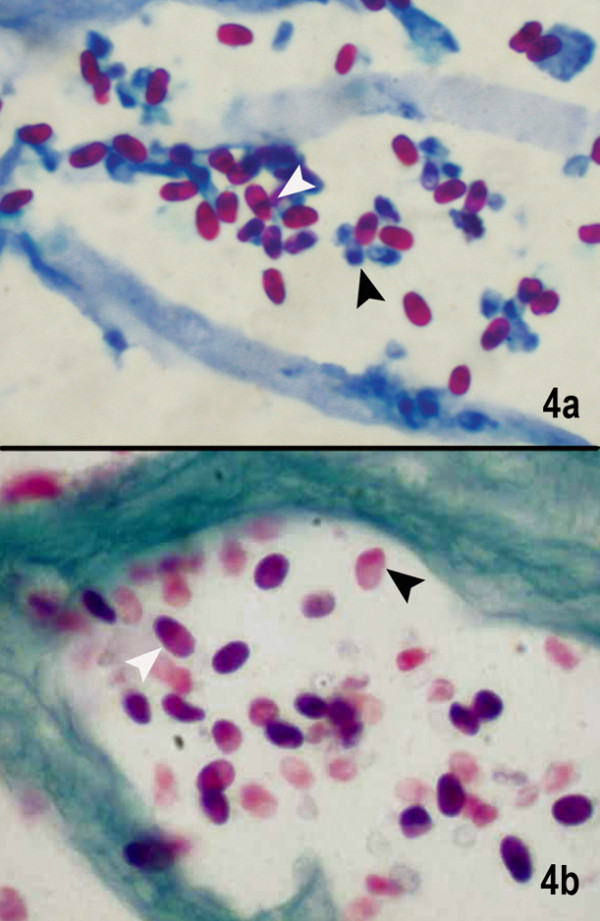
**(a) **Microsporidal spores are seen as well defined oval reddish bodies with a dark staining of the narrow end of the spore (black) or a waistband (white) closer to the tip of narrow end. Also seen are the unstained blue spores which possibly are immature or degenerating spores (1% acid fast stain, × 1000).(b) The spores are well delineated as purplish pink egg-shaped spores with a darker staining of the tip (white arrow). Even the degenerating spores show the darkly staining tip (black arrow) Gram's chromotrope stain (× 1000)

The spores appeared bright red against a bluish background. Some spores did not take up the stain and appeared blue, but all of them showed a thick band (arrowhead) like nucleus at one pole (Fig. 4a). This stain helps in enhanced detection of the spores as bacteria and other tissue structures appear blue. Here the spores could be identified even in low magnification.

### Gram's chromotrope stain

Spores in the chromotrope-stained smears appeared purple to pink, oval to round in shape, with the characteristic belt like strip in the middle (arrowhead), which was darker compared to the rest of the spore (Fig. 4b). Although a few spores did not take up the stain, the internal morphology could be appreciated, and all the spores appeared thick walled. The green background ensured a good contrast and the spores could be identified even in low magnification.

## Discussion

Definitive diagnosis of microsporidiosis has often depended upon microscopic detection of the spores in clinical samples. The spores appear as oval to round and posses a coiled filament, the number and arrangement of these coils vary among genera and species and these can be identified under transmission electron microscopy (TEM) [9,13]. However, it is time consuming, expensive and requires a great deal of expertise and is believed to be less sensitive than desired. Detection and characterization by molecular methods like PCR are still in their infancy in our country and a flexible diagnostic technique has never been more important than the present in view of increasing reports of microsporidiosis in India [14-16]. In view of the rarity of these cases and the possibility of an increase in such cases in future, we deemed it useful to evaluate the stains used in our laboratory for the identification of microsporidia.

Though cytologic methods are preferred for monitoring therapy as well as for better visualization of microsporidial spores due to less background debris, in cases where they cannot be used or smears are negative, histologic tissue examination are employed. We have earlier reported calcofluor white and modified acid fast stain to be the most useful stains for the diagnosis of microsporidial keratitis in corneal scrapings [17], but we wanted to know if these results also applied for tissue sections of patients who underwent penetrating keratoplasty. Histopathological examination of the corneal button has been reported to be instrumental in documenting the first case of ocular microsporidiosis [8] which revealed numerous oval bodies measuring 3–4 microns, that were weakly positive with H&E, PAS and Gram stain. Subsequently, it was recognized that the polar PAS-staining granule at the anterior end of mature spores is diagnostic for microsporidia [18]. In our experience too, histopathological confirmation with 1% acid fast stain after detection of spores on H & E stained section, of the first case of stromal keratitis due to microsporidia helped increase confidence in the detection of the spores in corneal scrapings. Though the use of acridine orange for the diagnosis of microsporidia has not been reported before we included it in our study as acridine orange has been reported to be useful in diagnosing Acanthamoeba, which is another protozoan parasite causing ocular infections. Moreover, we wanted to evaluate the entire spectrum of stains available in our laboratory that were commonly used in the diagnosis of microbial keratitis.

Based on our observations on tissue sections we believe that 1% acid fast stain, Gram's chromotrope stain and GMS stain have several advantages over other stains and therefore suggest the use of these in providing a reliable diagnosis of microsporidia. The characteristic waist band can be easily observed, and the good contrast achieved helps distinguish the spores from inflammatory debris. However, unlike 1% acid fast stain, GMS is not considered cost effective and Gram's chromotrope technique has not been standardized in most laboratories in our country. We would also like to highlight that classically PAS stain is used to bring out the "polar granule", which is not seen very well with other stains, which highlight the waist band. Unlike corneal scrapings [17], we observed that Calcofluor white and acridine orange were less suitable for microsporidia detection in paraffin sections mostly due to the background noise. Also, CFW being a wet preparation, unlike acid fast and Grams chromotrope stain and hence may pose problems in archiving of slides. However the variable staining of different stages of microsporidia within the same preparation should be kept in mind while interpreting the smears or sections.

Similar to cytologic diagnosis of *Helicobacter pylori *[19] and microsporidia by imprint smears of intestinal biopsies [6], the same could be attempted with corneal button tissues for diagnosing microsporidia. The imprint smears provide good cytologic details and are devoid of processing artifacts of the tissue, and do not alter the quality of the tissue which can subsequently be processed for histologic studies. A correlation of cytological and histopathological data wherever possible, helps in making a definitive diagnosis as well as rules out false positive results. We would also like to highlight that the spore size depends on the species involved and some species could have a predilection to particular sites/tissues. We have earlier reported the largest case series in the world on microsporidial epithelial keratoconjunctivitis [20]. We observed that corneal scrapings from patients with superficial keratoconjunctivitis showed much smaller spores (1–3 μm) compared to those observed in tissues (3–5 μm) in cases of stromal keratitis. The present series included cases of stromal keratitis, which is mostly reported by *Nosema *spp. and therefore despite the tissue processing which is known to cause 30–35% shrinkage of tissues, the spores appear larger than those observed in corneal scrapings from keratoconjunctivitis patients. This should be kept in mind while making a diagnosis of microsporidal keratitis. However, the results of this study can also help identify mirosporidia in corneal scrapings as well.

Using routine staining techniques such as H&E, only highly experienced pathologists have reliably and consistently identified microsporidia in formalin-fixed, paraffin embedded tissue sections [21]. Recognition of these cells on H & E staining should prompt the observer to request for special stains to confirm the diagnosis. In a study comparing special stains for the diagnosis of microsporidia, ie H & E, modified trichrome, Warthin Starry and Gram stain, modified trichrome was reported as the most effective [22]. In our series we were able to confirm the diagnosis using 1 % acid fast stain. In addition, though Gram's chromotrope stain is not routinely used, it can be easily standardized in any laboratory. In previous case reports of ocular microsporidiosis, diagnosis has been made using Gram, Giemsa, Uvitex B, PAS, GMS and modified trichrome stains [3,16].

Use of at least 2 or more staining methods is advisable to confirm the diagnosis specially when extremely scanty microsporidial spores are present in clinical samples to rule out false positive results. These observations we believe would help us to identify more number of clinically suspected cases wherein the corneal/conjunctival scrapings have failed to reveal organisms and a diagnostic biopsy may be indicated. However a limitation in our study is that speciation of our tissue sections was not carried out which could have helped us to further understand the differential staining patterns seen in our cases. We are still in the process of understanding this rare entity and our further ongoing study using molecular methods will allow us to determine the species of microsporida causing ocular infections. Further studies like electron microscopy, culture and immunofluorescence assays or polymerase chain reaction (PCR) with species-specific primers, whenever feasible should be considered to establish a precise diagnosis along with species identification [3,9]. High index of suspicion, use of 2 or more stains even in the absence of inflammatory response, and an eye for detail is warranted to increase the identification of this organism which is now being reported in many tissues of the body. Even though the cornea is avascular tissue with unique features, we believe that these observations could still be useful in identification of microsporidia in other tissues.

## Conclusion

We believe that Gram's chromotrope and 1% acid fast stains are dependable, cost effective methods of diagnosing microsporidiosis in ocular tissues. Molecular methods may complement the morphological diagnosis and aid in species identification of microsporidia.

## Competing interests

The author(s) declare that they have no competing interests.

## Authors' contributions

JJ carried out the experiments, collected data, participated in its design and drafting of the manuscript. GKV was involved in designing the experiments, carrying out the analysis and drafting the manuscript. PG provided the clinical information and edited the manuscript. SS participated in its design, and edited the manuscript. All authors read and approved the final manuscript.

## Pre-publication history

The pre-publication history for this paper can be accessed here:



## Figures and Tables

**Table 1:** Details of four patients presenting with microsporidial keratitis

**S.No**	**Age/Sex**	**Duration of Symptoms**	**History of trauma (Yes/no)**	**Presenting features**
1	2/M	l.5 months	no	stromal oedema, anterior stromal infiltrate
2	40/F	7 months	no	stromal infiltrate, endothelial exudates
3	37/M	4 years	Yes (fingernail)	Corneal opacity
4	70/F	6 months	Yes (grass)	deep stromal infiltrates, endothelial exudates

M – male

F – female

**Table 2:** Outline of stains used in histopathological evaluation

**S.No**	**Stain**	**Morphological features**	**Remarks**
1	Hematoxylin and Eosin	pale pink, ill-defined morphology	Most common tissue stain, should raise the suspicion of microsporidia. Appearance may resemble yeasts, needs confirmation by other stains
2	PAS	pink to purple in colour, ill defined morphology	Poorly stained, but the pink dot at the pole could suggest microsporidia. Not confirmatory
3	Giemsa	stain not taken up be cell wall, appears blue,	Stains the organism blue, but does not stand out against the blue background with also shows granular debris Easy to perform
4	GMS	oval to round, brown in colour, internal band is easily spotted, background green	Good stain, highlights the organism again the background under low magnification. Expensive reagents, time consuming
5	CFW	blue, oval, diffusely fluorescing spores, sometimes only spore wall fluoresced	Easy for experienced observer, but can be confused for yeasts. Produces high background noise Cannot be done without a fluorescence microscope
6	Acridine orange	oval, refractile, yellowish-green to orange fluorescence, no demarcation from the background	Easy for experienced observer, but can be confused for yeasts. Produces high background noise Cannot be done without a fluorescence microscope
7	Gram	gram variable, waist band visible, poor contrast from background, plenty of inflammatory debris	Economical, easy to perform. Variable staining, high background noise is a limitation. Microbiologists find it familiar and easy to extrapolate with cytologic findings
8	Masson's Trichrome	spores appeared red against a blue background, ill-defined internal morphology	High background noise, difficult to standardize the stain. Pathologists familiarity more than microbiologists
9	1% acid fast	oval, bright red with a darkly stained belt across the spore, visible against a blue background	Good contrast, easy interpretation, less time and economical. Unstained forms can also be found
10	Gram's chromotrope	spores appear purple to pink against green background, characteristic belt visible	Provides good contrast, easy to detect the organism under lower magnification. Has the added advantage of Grams and Massons trichrome, less time
